# Challenges of conducting research in long-term care facilities: a systematic review

**DOI:** 10.1186/s12877-018-0934-9

**Published:** 2018-10-12

**Authors:** Helen R Lam, Selina Chow, Kate Taylor, Ronald Chow, Henry Lam, Katija Bonin, Leigha Rowbottom, Nathan Herrmann

**Affiliations:** 1 Sunnybrook Health Sciences Centre, University of Toronto, 2075 Bayview Avenue, Toronto, ON M4N 3M5 Canada; 2 Division of Geriatric Psychiatry, Sunnybrook Health Sciences Centre, University of Toronto, 2075 Bayview Avenue, Room FG19, Toronto, ON M4N 3M5 Canada

**Keywords:** Research, Clinical trials, Challenges, Barriers, Nursing homes, Long-term care, Long term care hospitals, Chronic care, Skilled-nursing facilities, Residences for senior citizens

## Abstract

**Background:**

The aim of this review is to describe the challenges and barriers to conducting research in long-term care facilities.

**Methods:**

A literature search was conducted in Ovid MEDLINE, Embase, Cochrane Central, PsycINFO and CINAHL. Keywords used included “long term care”, “nursing home”, “research”, “trial”, “challenge” and “barrier”, etc. Resulting references were screened in order to identify relevant studies that reported on challenges derived from first-hand experience of empirical research studies. Challenges were summarized and synthesized.

**Results:**

Of 1723 references, 39 articles were selected for inclusion. To facilitate understanding we proposed a classification framework of 8 main themes to categorize the research challenges presented in the 39 studies, relating to the characteristics of facility/owner/administrator, resident, staff caregiver, family caregiver, investigator, ethical or legal concerns, methodology, and budgetary considerations.

**Conclusions:**

Conducting research in long-term care facilities is full of challenges which can be categorized into 8 main themes. Investigators should be aware of all these challenges and specifically address them when planning their studies. Stakeholders should be involved from an early stage and flexibility should be built into both the methodology and research budget.

**Electronic supplementary material:**

The online version of this article (10.1186/s12877-018-0934-9) contains supplementary material, which is available to authorized users.

## Background

As human life expectancy continues to increase, particularly in developed countries, more people will be living longer lives while being afflicted with chronic illnesses, multiple co-morbidities, and functional deficits [1–4]. As a result of the aging population, many individuals will require placement in long-term care (LTC) facilities [5, 6]. In the USA as of 2010, 1.2 million people or 3.1% of those aged 65 and over lived in a skilled-nursing facility, while in Canada as of 2011, 4.5% of those in the same demographic, yielding a total of 224,000 people lived in a ‘nursing home’ or ‘LTC hospital’ (LTC may be called skilled-nursing facilities, nursing homes, LTC hospitals, chronic care or residencies for senior citizens) [7, 8]. Thus, research aimed at improving geriatric medicine and the quality of care in LTC has become increasingly important. Despite this need, research in LTC settings, considered by some authors as a ‘highly unstable environment’ due to high attrition and turnover of staff or residents, as well as unpredictable external regulatory changes, remains challenging for various reasons [5, 9].

The challenges of conducting research in LTC facilities and/or on older adults have been described in a small number of reviews. Some of these focused on older adults as research subjects, but were not necessarily in LTC settings. For example, a Canadian guideline by Slaughter et al. reported on consent and assent of dementia patients as research subjects but this was not limited to LTC settings [2]. Another study discussed issues pertaining to intervention research in LTC, schools and critical care [9]. McMurdo et al. suggested potential improvements to recruitment of the elderly for research [10], and an article by Schulz discussed barriers to caregiver intervention research in various settings [11].

There have also been reviews focused on research challenges in LTC settings. A number of studies summarized ethical challenges [12–14], while others discussed the difficulties of studying a particular condition in LTC, such as falls, delirium and incontinence [15–17]. Ruckdeschel et al. targeted issues on gaining LTC staff support [18], while Reed et al. discussed the issue of research governance in LTC [19]. An article by Maas et al. in 2002 was a more extensive review, covering various challenges of nursing research in LTC, but it was not a systematic review and did not use a comprehensive literature search strategy [3]. We searched the PROSPERO website to check for existing systematic review protocols on this topic and found none [20]. The purpose of this study was to provide an updated and comprehensive systematic review of the challenges of conducting research in LTC facilities as reported in empirical research studies. By organizing these challenges into themes, we hope to provide investigators with a framework for anticipating and dealing with these challenges in order to facilitate research in LTC.

## Methods

A literature search was conducted in Ovid MEDLINE (1946 to June 2017, week 4), Embase Classic & Embase (1947 to 2017, week 27), Cochrane Central Register of Controlled Trials (May 2017), PsycINFO (1806 to June 2017, week 4) and Ebsco CINAHL (1981 to Jul 5 2017). Keywords such as ‘long-term care’, ‘nursing home’, ‘research’, ‘trial’, ‘challenge’, ‘barrier’, ‘issue’, ‘recruitment’, and ‘difficulty’, etc., were used to retrieve relevant studies which explicitly reported on challenges or barriers encountered during LTC research (see Additional file 1 for complete database search strategies). The searches were limited to English language and human subjects only. The PRISMA checklist was used to validate the research process [21]. (Additional file 2: PRISMA Checklist).

**Fig. 1:**
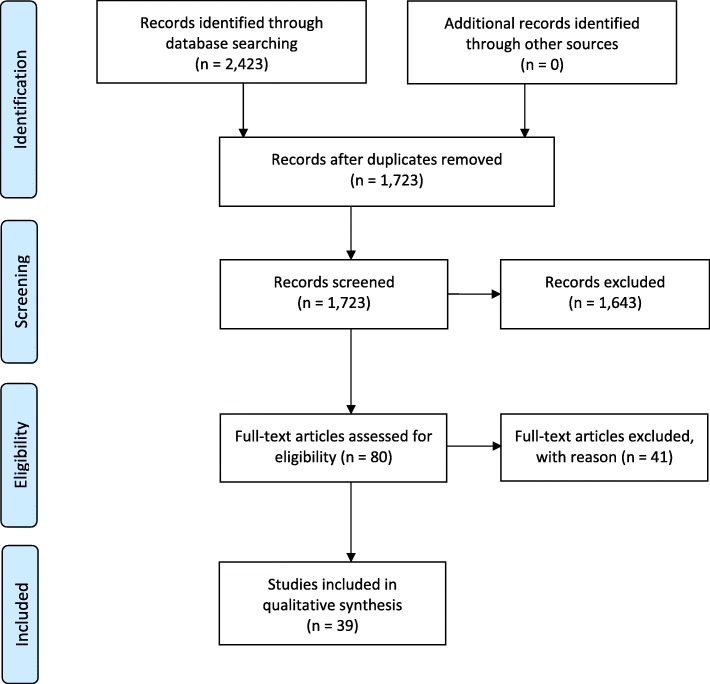
PRISMA Research Process Flow Diagram

The titles and abstracts of the search results were screened independently by two authors (HRL, KT) with discrepancies discussed and resolved. Articles were eligible for full-text screening if the title and/or abstract mentioned research and challenges, in addition to “long-term care”, “LTC”, “nursing home” or “care home”. Full-texts were screened for inclusion by 3 authors (HRL, SC, KT) with disagreements resolved by discussion. Articles were included in this systematic review if they reported on challenges or barriers experienced during empirical research studies in LTC or a nursing home setting. Research studies that made use of previously collected or administrative data were also acceptable if they satisfied other criteria. Studies were excluded if they were secondary studies such as narrative reviews, systematic reviews, guidelines, expert commentaries, or were editorials, letters, conference abstracts, unfinished studies, research protocols, about patients living at home or receiving home care, or non-English (Fig. 1).

The primary information of interest was the challenges and barriers experienced by the investigators conducting research in a LTC setting. A text-tagging software QDA Miner Lite and a thematic synthesis approach was employed to assist in summarizing the main challenges that were discussed in the studies [22, 23]. We imported the full texts of all 39 included studies into the software. Three authors (HRL, SC, HL) carefully read through the full texts and performed line-by-line tagging of the sections within the studies where authors described actual research challenges they had experienced in LTC research. Patterns were recognized and initial categories (descriptive themes) of challenges were identified and established by the same group after discussion to resolve disagreements. The texts were re-coded according to the descriptive themes. We then revised and consolidated the descriptive themes into a final hierarchy of analytical themes which were approved by all co-authors. Coding of texts was re-done once again according to the final categorization.

## Results

The literature search yielded 1723 references, of which 80 articles were selected for full-text screening as specified by the inclusion criteria, and ultimately 39 were included in this review (Fig. 1). They were all reports derived from actual experience of empirical research such as clinical trials, observational studies, surveys or epidemiological studies.

Of the 39 papers included in this review, 20 were based on studies conducted in the United States [24–43], 15 were based on those from the United Kingdom [44–58], and 1 study from each of Australia [59], New Zealand [60], France [61] and Norway [62]. Most of the 39 papers reported on challenges experienced in research with LTC residents as research subjects. A smaller number (4) of papers noted challenges encountered while studying subjects such as LTC facilities, LTC staff, or family members (see Table 1).

**Table 1:** Research Challenges in LTC Noted by Included Studies

References	Subjects of Primary Research (F: Facilities, FM: Family members, R: Residents, S: Staff)	Type of Challenge						Methodological	Budgetary
		Facility/ Owner/ Administrator	Resident	Staff Caregiver	Family Caregiver	Investigator	Ethical/ Legal		
Zapka J et al., 2014 [24]	R	✓		✓	✓		✓		
Garcia C et al., 2013 [25]	R	✓		✓		✓	✓		✓
Tilden VP et al., 2013 [26]	F, S	✓							
Ersek M et al., 2012 [27]	R	✓	✓					✓	
Greenspan S et al., 2012 [28]	R	✓	✓	✓			✓		
Van Ness PH et al., 2012 [29]	R	✓	✓					✓	
Hickman SE et al., 2008 [30]	R	✓					✓		
Quinn CC et al., 2008 [31]	R		✓	✓					
Gismondi PM et al., 2005 [32]	R	✓	✓	✓	✓		✓		✓
Decker CL et al., 2004 [33]	R		✓		✓		✓	✓	
Mentes JC et al., 2002 [34]	R			✓					
Snyder M et al., 2001 [35]	R		✓	✓					
Cohen-Mansfield J et al., 2002 [36]	R		✓						
Sherrell K et al., 1997 [37]	R	✓	✓			✓		✓	
Phillips LR et al., 1995 [38]	R			✓					
Williams SG et al., 1993 [39]	R		✓		✓		✓		
Cassel CK et al., 1988 [40]	R		✓				✓	✓	
Cohen-Mansfield J et al., 1988 [41]	R		✓						
Lipsitz LA et al., 1987 [42]	R		✓	✓			✓		
Palumbo FB et al., 1987 [43]	R	✓					✓		
Simpson P et al., 2017 [44]	FM, R		✓		✓				
Froggatt K et al., 2016 [45]	R	✓							
Jenkins C et al., 2016 [46]	S	✓		✓			✓	✓	
Tzouvara V et al., 2016 [47]	R	✓		✓					
Shepherd V et al., 2015 [48]	R	✓		✓		✓	✓	✓	
Davies SL et al., 2014 [49]	F, S	✓		✓					✓
Whelan PJ et al., 2013 [50]	R		✓						
Wood F et al., 2013 [51]	R		✓	✓	✓				
Goodman C et al., 2011 [52]	R	✓	✓	✓			✓		✓
Lasseter G et al., 2011 [53]	R		✓						✓
Hall S et al., 2009 [54]	R		✓						
Zermansky AG et al., 2007 [55]	R	✓	✓	✓				✓	✓
Hart E et al., 2005 [56]	R							✓	
Hubbard G et al., 2003 [57]	R		✓			✓			
Shah A, 1998 [58]	R			✓			✓		
Murfield J et al., 2011 [59]	R	✓	✓			✓		✓	
Peri K et al., 2008 [60]	R			✓				✓	
Bloch F et al., 2014 [61]	R		✓						
Heggestad AKT et al., 2013 [62]	R		✓	✓	✓		✓		

With the help of a thematic synthesis approach we created a framework of 8 main themes to categorize the challenges reported in the 39 studies: facility/owner/administrator factors, resident factors, staff caregiver factors, family caregiver factors, investigator factors, ethical/legal factors, methodological factors and budgetary factors (Table 1). We then described what the 8 main themes represent, and which and how many studies noted challenges that fall under a particular theme.

We summarized below the findings of the 39 studies under each of the eight themes.

### Facility/owner/administrator factors

A total of 18 studies reported challenges that could be categorized under this theme [24–30, 32, 37, 43, 45–49, 52, 55, 59]. Some of the major issues included within this theme were: recruitment and retention of LTC facilities, owners or administrators unfamiliar with research, not interested or feel threatened.

A major challenge reported by authors was the recruitment and retention of LTC facilities to participate in clinical research, as many facilities were reluctant to do so [27, 46]. Participation in research was not a common experience for most LTC facilities [30]. Hickman et al. reported that just over half (53%) of the facilities they surveyed had previously participated in research, with experiences ranging from a single resident enrolled in an off-site study to larger scale studies involving modifications to the physical environment [30]. Some LTC owners and/or administrators refused to allow research to be conducted in their facilities, while others were prohibited from participating due to corporate policies [24, 49]. Additionally, some were reluctant to take part because their facilities would be subjected to more government inspection as a result [24]. In general, large, privately-owned LTC chains have less flexibility when there is a corporate-wide policy that prohibits research, whereas small independent non-profit facilities may be restricted from participating in research due to their limited resources [29]. In fact, Gismondi et al. reported a low participation rate of 20% from large corporations, and an even lower rate of 14% from independent facilities [32]. Moreover, recruitment of LTC facilities can be time consuming, ranging from 8 to 13 months [48, 49].

Changes in ownership posed another challenge to conducting research in LTC facilities [25, 46]. In a study conducted by Sherrell et al., 80% of nursing homes had changed their names by the time the follow-up study was administered [37]. Retention and attrition of LTC facilities was a problem, especially in longitudinal studies and among facilities with logistical deficiencies and high personnel turnover [26].

Administrators’ personal views regarding the value of research were often mixed [30]. They had varied experiences and education backgrounds and many were suspicious of the investigators’ motives for entering the facility [37, 52]. Some might have felt threatened by an outsider investigating their service, or felt that research was uninteresting and that the results would not be applicable to their facilities [30]. There were also concerns that research might not reflect a true picture of the facility, with the potential risk of resident abuse being observed and reported to the licensing agency [25, 30]. Some administrators claimed to be too busy to return calls or messages from investigators, or simply declined without any explanation [25, 43, 47]. In contrast, some LTC facility owners only consented to research studies to prevent jeopardizing government funding [37].

Investigators also face logistical challenges. In the experiences of several investigators, experimental treatments or placebos were not always administered according to plan during the investigators’ absence [24, 49, 59]. Medical and hospitalization records might not be easily accessible, due to little uniformity in the collection of health records [28, 55]. Additionally, if confidential interviews are held as part of a research study, an appropriate environment may not be available in a LTC facility due to space constraints [45, 59].

### Resident factors

There were 24 studies which reported challenges that fall under this theme [27–29, 31–33, 35–37, 39–42, 44, 50–55, 57, 59, 61, 62]. Some major issues included in this theme were: recruitment, consent, residents prefer treatment over placebo, and attrition.

LTC residents are a highly heterogeneous group with different backgrounds and health conditions. As a result, their recruitment, retention, and ability to consent and participate are all potentially problematic. Some authors reported the resident recruitment phase to be the most difficult part of research in LTC, especially among residents with dementia, with a success rate of 0% to 46% in one study [53]. In another study, Bloch et al. reported that only 44% of LTC residents were willing to participate in trials, in hopes that it would provide personal benefit [61].

Participants often preferred to receive the treatment rather than the placebo, creating bias and making randomization difficult [40, 59]. On the other hand, many residents were not even willing to participate, as they did not trust the motives of the investigators and disliked the interruption to their daily routine [28, 54]. On average, low-risk observational studies experienced higher recruitment rates, whereas pharmacologic studies involving residents with frail conditions had much lower rates [36, 42, 51]. Residents often stated the following reasons for refusal: “I am too old for this type of experiment”; “I am afraid of taking an ineffective drug”; “I am already using a lot of drugs”; “I don’t want to be a guinea pig” [61]. Some residents were afraid of invasive interventions, such as venipuncture or urethral catheterization [42]. Finally, recruitment within minority groups was even less successful due to cultures that might not value research participation, and the additional cost of hiring interpreters, resulting in minorities being under-represented in LTC research [24, 32, 33].

Many residents did not consent to participate in research, as they did not see how they could personally benefit from the study within their lifetime [28, 33, 36, 42, 61]. Others perceived research as an invasion of privacy, afraid that confidentiality would not be maintained [39–41, 54, 62]. The concern of lack of confidentiality was attributed to the fact that some residents shared a room with other residents, and the doors were likely kept open during the process [33, 54]. Many also felt uncomfortable with certain research topics, including sexuality, dementia and death [44, 52].

Many residents who were sick or cognitively impaired may not have had the capacity to give consent or to effectively communicate verbally [37, 44, 57]. Despite this, investigators included cognitively impaired residents, because most residents in LTC facilities typically have some degree of cognitive impairment [27, 55]. As such, recruitment requirements needed to be adjusted according to the level of disability in each facility [42].

Obtaining informed consent from LTC residents, including cognitively impaired and emotionally unstable candidates, was described as difficult and time consuming [28, 40, 50, 54, 59]. Residents’ ability to consent could change during the research period, and some were excluded unnecessarily due to incorrect evaluations of their ability to consent [40, 51, 62]. Some residents lost interest during the lengthy consent process and others felt pressured by the investigators [28, 32]. Obtaining consent for research on short-stay subjects in LTC facilities was problematic due to the time constraints [31]. (The challenge of obtaining consent from residents with cognitive impairment is also listed and elaborated under Ethical/Legal Factors).

Even when investigators successfully recruited enough participants in a study, retention and attrition is a common issue, due to study withdrawal, high mortality, comorbidity, hospitalizations and transfers to another facility [29, 33, 51, 55]. In particular, some residents could not complete the study because of mood or behavioral problems [35]. For example, participants could become aggressive or agitated, or could refuse to take part because they wish to avoid disruptions to their daily routine [35]. Even if they continue to participate despite being in an agitated state, the data obtained may not be accurate [57]. (Attrition is also noted as an issue under the Methodological Factors theme).

### Staff caregiver factors

There were 19 studies which reported challenges that could be categorized under this theme [24, 25, 28, 31, 32, 34–36, 38, 42, 46–49, 51, 52, 55, 58, 60, 62]. Some major issues included in this theme were: time constraint, turnover, low education level, and uncooperative attending physicians.

Staff time constraint was a major obstacle in arranging for training and execution of research [28, 31, 34, 35, 42, 46, 47, 49, 58]. In order to participate in research, LTC staff are often required to attend meetings, accompany investigators to see residents, help contact families, assist in obtaining consent, and take part in the intervention, all in addition to their regular responsibilities [24, 25, 28, 34, 52]. Initial staff enthusiasm might wear out quickly once they realize the amount of time commitment required [34, 51]. Peri et al. found that staff willingness was the main factor affecting recruitment rates [60]. If the nurses do agree to participate in a study, their assessments of residents may not necessarily be reliable, as not all nurses may be aware of the details of residents’ conditions nor do they correctly recommend appropriate candidates for research [32, 62].

Another concern is high staff turnover, making it harder to carry out studies [24, 28, 34, 46]. In our experience this is a particular concern when nursing assessments are primary outcome measures, and changes in the main rater may compromise validity.

According to Phillips et al., power dynamics in LTC facilities could be complex and consequently, non-professional staff caregivers might have unexpected influences on research [38]. For instance, interventions may not have been carried out as planned when investigators were not on-site [38]. As well, the education levels among non-professional caregivers can be low and some may be hesitant to serve as witnesses to the consent process [32, 38].

Medical staff can pose other challenges. Attending physicians at a facility might be concerned about ‘outsider’ physicians being involved with ‘their residents’ [24]. There may also be billing or compensation issues [24, 31, 51]. Private physicians might not have returned calls or met with the investigators, or refused to co-operate in providing medical records [31, 48, 55].

### Family caregiver factors

A total of 7 studies noted challenges that fall under this theme [24, 32, 33, 39, 44, 51, 62]. Some major issues included in this theme were: constraint of their own physical condition, schedule and education level; see no benefits to the residents, view research as invasion of privacy.

Seeking consent and assent from family members was challenging due to their own physical conditions, work schedules, visit times or education levels [24, 32, 33, 51]. While many family members believed that elderly residents should take part in studies, their opinions changed when the subjects had dementia or when they felt that there was an invasion of privacy [39]. Some believed that residents should receive financial compensation for participating in research [39]. Others had reservations about relatives participating in clinical studies, even if the latter had agreed, as they felt that there was nothing for the residents to gain or that the topic of study (e.g. sexuality) was inappropriate [24, 44, 51, 62]. It was also difficult to have family caregivers attend regular meetings during the research period [33].

### Investigator factors

A total of 5 studies reported challenges that fall under this theme [25, 37, 48, 57, 59]. Some major issues included in this theme were: principal investigators (PIs) perceived as threat or outsiders, and high turnover of research assistants (RAs).

In certain countries including the United Kingdom, a PI is required to be available on-site during the intervention(s) [48]. PIs from outside the institutions can be seen as ‘outsiders’ or threats to the LTC facilities [25, 37]. For PIs and RAs who work primarily in other institutions, travel to the facility and communication with staff in the LTC can be significant potential barriers. Examples of communication problems included phone calls or letters from external PIs/RAs being ignored by LTC staff [25]. PIs and RAs also need to have special verbal and non-verbal communication skills to collect data from subjects with cognitive impairments, which can add to the cost due to the additional training required [57].

The cost to hire enough RAs and their high turnover are other barriers to research in LTC facilities, especially since RAs are essential in overseeing the logistics of the research process, in order to avoid over-dependence on the facilities’ manpower [59].

### Ethical/legal factors

A total of 15 studies reported challenges that fall under this theme [24, 25, 28, 30, 32, 33, 39, 40, 42, 43, 46, 48, 52, 58, 62]. Some major issues included in this theme were: obtaining consent legally and ethically, withholding of likely effective treatments, and lack of IRB or REB familiar with LTC.

Ethical issues were significant challenges noted by many investigators. Consenting individuals who lack the physical and mental capacity to participate in a study, especially when discomfort, pain, psychological distress or physical risk are potential side-effects, was seen as potentially problematic by some investigators [30, 42, 46, 48]. It was also an ethical dilemma when investigators incidentally discovered any sub-standard or inadequate treatment of residents in the LTC facilities during the research process [25, 33, 40, 58]. Furthermore, having a placebo group was another ethical issue when patients were knowingly withheld from a treatment that was known to improve outcomes [28]. Finally, research on subjects with dementia posed several issues, as many patients may have been unaware of their diagnosis and the concern that LTC administration might use the study’s findings as a reason to raise fees for those individual patients [52, 62]. (Obtaining consent from cognitively impaired residents is also listed as a challenge under Resident Factors).

In addition to ethical challenges, researchers also encountered legal concerns when conducting their studies in LTC facilities [30, 52]. For instance while ethics committees or institutional review boards (IRBs) are required for approval of research projects, many LTC facilities lack such an oversight body [28, 30, 39, 40, 43, 62]. Since many LTC facilities do not have internal IRBs, they have to rely on external ones, which can be time consuming and troublesome, as the members of external boards may lack first-hand knowledge about LTC facilities [24, 25, 30, 32, 48, 58]. These regulatory challenges have been shown to affect LTC facilities’ decision to participate in research [52].

### Methodological factors

There were 11 studies which described challenges that can be categorized under this theme [27, 29, 33, 37, 40, 46, 48, 55, 56, 59, 60]. Some major issues included in this theme were: randomization and finding suitable outcome measures.

Clinical trials were described as being more complex and time consuming to set up than observational studies in LTC settings [48]. Attrition due to resident turnover and mortality posed a challenge to study design [27, 29, 37]. (Attrition is also discussed as an issue under the theme Resident Factors). Randomization of individual residents was also troublesome in clinical trials, as some residents did not understand the importance of randomization and preferred to be subjects in the active treatment groups rather than controls [40].

Some investigators tried to overcome the problem of randomization with cluster randomization, where entire institutions were randomized to either the treatment or placebo group [27, 46]. However, a larger sample size is required to achieve adequate statistical power for cluster randomization, in order to account for the correlation among subjects within a cluster [29, 46]. As a result, several studies attempted to stratify multiple LTC facilities by type, size, and quality of care, which required more resources and complicated the standardization of study protocols [27, 29, 33, 55].

Another challenge of conducting research in LTC facilities was in selecting a suitable outcome measure customized for the LTC environment and its specific population [27, 33, 56, 59, 60]. While there are increasing numbers of rating scales that have been developed specifically with LTC environment in mind (e.g. the Neuropsychiatric Inventory- Nursing Home version [63]), due to the residents’ varying cognitive and functional abilities as well as their ability to communicate, many instruments and scales designed for non-LTC populations needed to be adapted before usage [27, 33].

### Budgetary factors

A total of 6 studies reported challenges related to this theme [25, 32, 49, 52, 53, 55] . The major issue of this theme was high costs.

The cost of conducting research in LTC facilities could be over three times higher than studies conducted in community settings [55]. The high cost of research was primarily attributed to the lengthy recruitment process of facilities, residents and staff [25, 32, 49, 52, 53]. Other incidental costs might need to be considered including approval costs from an external REB, travel expenses to and from the LTC for investigator and RAs, and specific training of LTC staff [32, 49, 52]. Other budgetary barriers not mentioned in the included studies but were known to us from our own research experience included costs for transportation of drugs and for use of the LTC pharmacy, as well as equipment and storage costs in the LTC. Finally, the research costs are even higher when interpreters are needed in LTC facilities with large non-English speaking populations [32].

## Discussion

Though lacking the attention that research in the acute care setting receives, research in LTC is important because the setting houses a population that suffers disproportionately from chronic diseases and functional deficits, consumes more medication, and has more complex health care needs than many groups [25, 40, 48, 61]. In the UK, for example, one third of the people with dementia live in LTC and approximately 80% of LTC residents have dementia, making LTC a logical location for dementia research [46]. Apart from benefits to the general population, research results from LTC are potentially more beneficial to LTC residents as they as a group are unique for their frailty and multiple-morbidity, and research results from other settings may not always be generalizable to them [55].

We think more education to residents and families about the unique advantages of LTC research in order to foster a culture of research study participation is needed. Immediate, direct benefits to participants may not frequently be obtainable, but numerous indirect benefits such as diversion from daily routine, opportunity to meet and interact with people, greater access to professional care, and to be able to feel useful and meaningful by making altruistic contribution, are not necessarily trivial to residents’ well-being [2]. While LTC facilities appear to be perfect ‘clinical laboratories’ with 24-h on-site staff, a controlled environment and long-staying residents, there are in fact many challenges that investigators face when conducting research in these institutions [38]. We have summarized the challenges and potential solutions in Table 2.

**Table 2:** Suggested Solutions to Research Challenges

Challenges	Specific Issues	Suggested Solutions
Facility/owner/administrator factors	Recruitment & retention of facilities	- PI develop long-standing relationship with LTC facility [24, 47] - Establish LTC research network [49] - Target facilities with history of research participation [47] - Provide continuing education opportunities to staff
	- Unfamiliar with research - Not interested - Feel threatened	- Improve education for owners and administrators [25, 26, 49] - Share research ownership [24, 47] - Include administration in study design and development
Resident factors	- Recruitment - Consent - Lack of trust - No personal benefit - Invasion of privacy	- Carefully designed, concise, and easy to understand recruitment materials [24, 43] - Assure privacy despite difficulties [43] - Emphasize to residents that their decision will not affect relationship with staff [42] - Offer financial compensation [42] - Develop flexible recruitment criteria [28] - Have trained home staff members do the initial approach for participation
	Prefer treatment over placebo	- Double-blinded, cluster randomization [27, 46] - Educate residents about the benefits of participation e.g. more intense monitoring
	Attrition	- Consider possibility of high attrition when calculating sample size
Staff caregiver factors	Time constraint & disruption to schedule	- Careful planning to minimize disruption - Offer financial compensation
	Turnover	- Recruitment of residents and study assessment carried out by investigators or RAs to reduce burden on LTC staff [28]
	Low education level	- Improve training on staff caregivers [49]
	Uncooperative attending physicians	- Offer a contract or letter of agreement [48] - Provide continuing education and online training [48]
Family caregiver factors	Constraint of their own physical condition, schedule, education level	- Carefully designed, concise, and easy to understand recruitment materials [24, 43] - Reduce travel/meeting time of family caregivers
	Sees no benefits to the resident	- Offer financial compensation [42] - Educate families about the benefits of participation e.g. more intense monitoring
	Invasion of privacy	- Assure privacy despite difficulties [43]
Investigator factors	Seen as a threat or an outsider	- PI develop long-standing relationship with LTC facility [24, 47] - Share research ownership [24, 47]
	High turnover of RAs	- Prioritize selection, training and support for RAs [34]
Ethical/legal factors	Obtaining consent legally and ethically	- Investigators should educate themselves about relevant regulations [32] - Establish standard procedures for obtaining consent
	Lack of internal REB that is familiar with LTC	- Collaborate with the LTC administration to determine which REB will be consulted
Methodological factors	Resident randomization	- Consider cluster randomization [3, 9] - Consider quasi-experimental design [3, 9]
	Outcome measures	- Use outcome measures designed specifically for LTC - Adapt outcome measures not specific to LTC if necessary [27, 33]
Budgetary factors	High cost	- Good planning and coordination to reduce costs - Budget cautiously with travel costs and site specific factors in mind

Since recruitment of facilities, staff, and residents, along with obtaining resident consent are major concerns, some authors suggest that these challenges can be reduced if PIs develop long-standing, respectful relationships with LTC facilities and share ownership of the studies [24, 47]. Other methods to increase recruitment include establishing LTC research networks, providing better research training for LTC staff, and inviting the administrator(s) to take an active role in research studies, including participation in study design and share research ownership [24–26, 47, 49]. It is also recommended that PIs target institutions with a history of successful research participation [47]. PIs should pay extra attention to the selection, retention, training and support of RAs, as they are crucial to the success of a research project [34]. Attending physicians’ participation can be encouraged by offering a formal contract, continuing education sessions and online training in preparation for the project [48], while better training should also be arranged for staff caregivers with lower education levels [49]. Disruption to the daily schedule of LTC facilities should be minimized with careful planning, or financial compensation can be offered to the staff affected.

In order to encourage resident and family consent, recruitment materials must be carefully designed, concise and easy to understand [24, 43]. Benefits of participation should be communicated to family caregivers. Travel and meeting times may be reduced to facilitate participation of family caregivers who might be constraint by their own physical condition, schedule and education level. Financial compensation to family caregiver may be another incentive to encourage participation [42]. Resident privacy must also be assured, although this may be difficult to maintain given the layout of certain facilities [43]. Likewise, residents and families should be informed that their decision to participate in a study will not affect their relationship with staff caregivers [42]. Trust of residents may also be improved by having trained staff caregivers with whom the residents are familiar do the initial approach for recruitment. As well, it is essential that recruitment requirements are flexible to the degree of disability in each facility, and some authors have suggested that monetary incentives can help increase resident recruitment [28, 42]. In order to counter some residents’ preference to be in the treatment rather than placebo group, cluster randomization where a whole institution or ward is assigned to the same group, and/or double-blinding where caregivers do not know which group residents are assigned, may be used [27, 46]. High attrition rate should be considered when calculating sample size.

Legal and ethical challenges are relatively universal across LTC facilities and investigators must educate themselves about all relevant local and national regulations before conducting research in LTC facilities [32]. Determining what body will act as the IRB for the study should be done in consultation with the LTC administration. Having standard operating procedures for obtaining informed consent is also highly recommended.

In regard to the methodology of research studies, while investigators should aim for rigorous designs using control groups, randomization and concurrent data collection, compromises such as quasi-experimental designs and cluster randomization can also be considered [3, 9]. Sample size calculations should include consideration of attrition due to withdrawals, death and transfers. Pre-specified rules can be designed for dealing with caregiver changes. Whenever possible, utilizing rating scales and outcome measures designed for LTC is preferable to adapting measures designed for community dwelling individuals. Finally, considering special budgetary considerations for performing the study in a LTC environment is highly recommended.

Many of the challenges and barriers to research described above would be similar to studying a frail elderly patient population in acute care or community samples. For example, in terms of recruiting sites from an acute hospital setting, in two recent larger scale clinical trials on subjects of variable age groups, 31% and 43% of hospitals contacted eventually participated [64, 65], and while higher than the LTC site recruitment figures quoted above, these are still low. In terms of under-recruitment and attrition of elderly subjects in clinical studies, this factor is certainly not unique to LTC and is evident in many health care settings [10, 66, 67]. For instance, recruitment rates for clinical studies involving the in community and hospital settings vary greatly, with examples ranging from figures as low as 3.4%, and as high as 89.9% [67–72]. Staff time constraint or staff non-cooperation as a barrier to research participation is also not exclusive to LTC. For example, they are common reasons for non-participation in research in hospitals or palliative care facilities as well [65, 73]. Methodological challenges are not limited to research in LTC. Setting up of clinical trials, achievement of proper randomization, and selection or development of suitable outcome measures are all common challenges found in health care research in various settings [74, 75]. Finally, funding or budgetary factors is a commonly reported challenge for health care research in any setting, though some authors believe it is particularly challenging in LTC [40, 76, 77]. Having noted these similarities, we still believe the LTC setting is unique. As stated above, the patient population in LTC is generally older and frailer cognitively and physically, with greater medical comorbidity and medication polypharmacy. LTC residents are “residents” and not “patients” – the LTC facility is their home, changing the context of the relationship between subject and investigator likely affecting recruitment. The LTC facilities are generally not connected with general hospitals and universities, and are therefore unfamiliar with a research culture. Administrators may see research participation as a way to raise the profile of their LTC, or alternatively, an inconvenient and disruptive distraction from the business of caring for their elderly residents. These differences are more than sufficient to require investigators’ attention if successful research is to be conducted in these unique settings.

There are some limitations to our study. We restricted our search to English language materials only, which may have led to bias against non-English papers which may be smaller scale studies, or studies with less remarkable results [78]. With our search strategies, we searched for explicit reports of challenges that contain any of the keywords such as ‘challenge’, ‘barrier’, ‘issue’, ‘factor’, ‘recruit’, ‘problem’, ‘attrition’, ‘difficult’, ‘dropout’, ‘retention’, etc. in the titles or abstracts (see Additional file 1 for complete search strategy). We did not scan through all empirical studies in LTC without these keywords to look for mention of challenges or possible solutions. While we believe the chance of missing papers focused on challenges or barriers without these keywords is low, this remains a potential limitation of our study. Finally, the thematic synthesis approach we used, as with any qualitative research method, involves a certain level of subjective judgement of the reviewers involved [22]. But despite all these limitations, we believe we are able to present a summary of the literature based on the actual experience of researchers who have conducted researches in LTC.

## Conclusions

This systematic review searched and summarized studies reporting challenges of conducting research in LTC facilities. With a thematic synthesis approach, we were able to categorize the challenges noted in the 39 studies into 8 main themes: facility/owner/administrator factors, resident factors, staff caregiver factors, family caregiver factors, investigator factors, ethical/legal factors, methodological factors and budgetary factors. We think this integrative framework of 8 main themes can assist readers in understanding the challenges reported in the literature to date.

Investigators should familiarize themselves with these challenges and attempt to address each barrier in advance through careful planning. Stakeholders including IRBs, LTC owners, administrators, staff representatives and physicians should be consulted at an early stage and have input into study design. Ideally, residents and family caregivers should also provide input and/or feedback. In this way, research in LTC becomes a partnership in which quality of care and quality of life can ultimately benefit.

## Additional files

Additional file 1: Search strategies used in Ovid MEDLINE, Embase, Cochrane Central Register of Controlled Trials, PsycInfo & CINAHL. (DOCX 15 kb)

Additional file 2: PRISMA checklist. (DOCX 25 kb)

## Abbreviations

IRB: Institutional review board
LTC: Long-term care
PI: Principal investigator
RA: Research assistant
REB: Research ethics board
