# Supplementary material for: Challenges of conducting research in long-term care facilities: a systematic review
Source: BMC Geriatr. 2018 Oct 12;18:242. doi: 10.1186/s12877-018-0934-9 (PMC6186062; doi:10.1186/s12877-018-0934-9)
Supplement: Supplementary file 1 — Search strategies used in Ovid MEDLINE, Embase, Cochrane Central Register of Controlled Trials, PsycInfo & CINAHL. (DOCX 15 kb) [file 12877_2018_934_MOESM1_ESM.docx]

**Additional file 1.** Database Search Strategies

Ovid MEDLINE <1946 to June Week 4 2017>
-------------------------------------------------------------------------------
1     exp Long-Term Care/ (24337)
2     long term care.mp. (32669)
3     exp Nursing Homes/ (36140)
4     (nursing home* or care home*).mp. (41293)
5     or/1-4 (69782)
6     research.ti. (186869)
7     trial*.ti. (214483)
8     (study or studies).ti. (1402438)
9     or/6-8 (1773742)
10     challenge*.ti,hf. (71692)
11     (accrual or accrued or nonaccrual).ti,hf. (427)
12     issue*.ti,hf. (54276)
13     factor*.ti,hf. (579118)
14     recruit*.ti,hf. (22291)
15     problem*.ti,hf. (177904)
16     barrier*.ti,hf. (37053)
17     attrition.ti,hf. (1509)
18     participat*.ti,hf. (28677)
19     approv*.ti,hf. (7498)
20     availab*.ti,hf. (129248)
21     difficult*.ti,hf. (25504)
22     (dropout or drop out).ti,hf. (1127)
23     retention.ti,hf. (25070)
24     (research adj2 ready).ti,hf. (16)
25     or/10-24 (1146952)
26     5 and 9 and 25 (479)
27     limit 26 to english language (427)

Embase Classic+Embase <1947 to 2017 Week 27>
--------------------------------------------------------------------------------
1     long term care/ (112590)
2     long term care.mp. (120811)
3     exp nursing home/ (48561)
4     (nursing home* or care home*).mp. (60758)
5     or/1-4 (171747)
6     research.ti. (238382)
7     trial*.ti. (311895)
8     (study or studies).ti. (1939058)
9     or/6-8 (2451472)
10     challenge*.ti,kw. (102057)
11     (accrual or accrued or nonaccrual).ti,kw. (829)
12     issue*.ti,kw. (76549)
13     factor*.ti,kw. (941955)
14     recruit*.ti,kw. (32006)
15     problem*.ti,kw. (215527)
16     barrier*.ti,kw. (66938)
17     attrition.ti,kw. (2539)
18     participat*.ti,kw. (41843)
19     approv*.ti,kw. (12876)
20     availab*.ti,kw. (94525)
21     difficult*.ti,kw. (37371)
22     (dropout or drop out).ti,kw. (2246)
23     retention.ti,kw. (39010)
24     (research adj2 ready).ti,kw. (28)
25     or/10-24 (1639626)
26     5 and 9 and 25 (1149)
27     limit 26 to english language (1062)

Cochrane Central Register of Controlled Trials <May 2017>
--------------------------------------------------------------------------------
1     exp Long-Term Care/ (1028)
2     long term care.mp. (4522)
3     exp Nursing Homes/ (1058)
4     (nursing home* or care home*).mp. (2943)
5     or/1-4 (7132)
6     research.ti. (5833)
7     trial*.ti. (198423)
8     (study or studies).ti. (188407)
9     or/6-8 (373110)
10     challenge*.ti,ab. (16066)
11     (accrual or accrued or nonaccrual).ti,ab. (2510)
12     issue*.ti. (637)
13     factor*.ti. (18077)
14     recruit*.ti. (1652)
15     problem*.ti. (3075)
16     barrier*.ti,ab. (7317)
17     attrition.ti,ab. (1533)
18     participat*.ti. (1832)
19     approv*.ti. (250)
20     availab*.ti. (966)
21     difficult*.ti. (1033)
22     (dropout or drop out).ti,ab. (8581)
23     retention.ti,ab. (9390)
24     (research adj2 ready).ti,ab. (3)
25     or/10-24 (68872)
26     5 and 9 and 25 (305)
27     limit 26 to english language (236)

PsycINFO <1806 to June Week 4 2017>:
--------------------------------------------------------------------------------
1     long term care/ (4403)
2     long term care.mp. (7708)
3     nursing homes/ (7740)
4     (nursing home* or care home*).mp. (12956)
5     or/1-4 (18650)
6     research.ti. (88474)
7     trial*.ti. (31145)
8     (study or studies).ti. (275363)
9     or/6-8 (388211)
10     challenge*.ti,ab,id. (168014)
11     (accrual or accrued or nonaccrual).ti,id. (72)
12     issue*.ti,id. (61881)
13     factor*.ti,id. (194667)
14     recruit*.ti,id. (6654)
15     problem*.ti,id. (134728)
16     barrier*.ti,id. (13215)
17     attrition.ti,id. (2256)
18     participat*.ti,id. (30856)
19     approv*.ti,id. (1898)
20     availab*.ti,id. (6366)
21     difficult*.ti,id. (21275)
22     (dropout or drop out).ti,id. (2778)
23     retention.ti,id. (15614)
24     (research adj2 ready).ti,id. (4)
25     or/10-24 (617046)
26     5 and 9 and 25 (397)
27     limit 26 to english language (355)

CINAHL Strategy<1981 to July 2017>:

| **Search ID#** | **Search Terms** | **Search Options** | **Results** |
| --- | --- | --- | --- |
| S26 | S5 AND S9 AND S25 | Limiters - English Language | 343 |
| S25 | S10 OR S11 OR S12 OR S13 OR S14 OR S15 OR S16 OR S17 OR S18 OR S19 OR S20 OR S21 OR S22 OR S23 OR S24 |  | 199,741 |
| S24 | TI (research N2 ready) |  | 29 |
| S23 | TI retention |  | 4,355 |
| S22 | TI (dropout or drop out) |  | 344 |
| S21 | TI difficult* |  | 6,797 |
| S20 | TI availab* |  | 6,003 |
| S19 | TI approv* |  | 6,021 |
| S18 | TI participat* |  | 11,063 |
| S17 | TI attrition |  | 485 |
| S16 | TI barrier* |  | 10,107 |
| S15 | TI problem* |  | 26,921 |
| S14 | TI recruit* |  | 5,854 |
| S13 | TI factor* |  | 59,959 |
| S12 | TI issue* |  | 40,489 |
| S11 | TI (accrual or accrued or nonaccrual) |  | 109 |
| S10 | TI challenge* |  | 27,152 |
| S9 | S6 OR S7 OR S8 |  | 294,747 |
| S8 | TI (study or studies) |  | 172,353 |
| S7 | TI trial* |  | 57,225 |
| S6 | TI research |  | 73,325 |
| S5 | S1 OR S2 OR S3 OR S4 |  | 56,251 |
| S4 | TX (nursing home* OR care home*) |  | 36,504 |
| S3 | (MH "Nursing Homes+") |  | 18,880 |
| S2 | TX long term care |  | 30,430 |
| S1 | (MH "Long Term Care") |  | 18,348 |
